# Towards the Development of Nonlinear Approaches to Discriminate AF from NSR Using a Single-Lead ECG

**DOI:** 10.3390/e22050531

**Published:** 2020-05-08

**Authors:** Jieun Lee, Yugene Guo, Vasanth Ravikumar, Elena G. Tolkacheva

**Affiliations:** 1 Department of Electrical and Computer Engineering, University of Minnesota-Twin Cities, Minneapolis, MN 55455, USA; leex4637@umn.edu (J.L.); ravik014@umn.edu (V.R.); 2 Department of Biochemistry, University of Minnesota-Twin Cities, Minneapolis, MN 55455, USA; guoxx947@umn.edu; 3 Department of Biomedical Engineering, University of Minnesota-Twin Cities, Minneapolis, MN 55455, USA

**Keywords:** paroxysmal AF discrimination, nonlinear dynamic method, time-delayed embedding, multiscale entropy, kurtosis

## Abstract

Paroxysmal atrial fibrillation (Paro. AF) is challenging to identify at the right moment. This disease is often undiagnosed using currently existing methods. Nonlinear analysis is gaining importance due to its capability to provide more insight into complex heart dynamics. The aim of this study is to use several recently developed nonlinear techniques to discriminate persistent AF (Pers. AF) from normal sinus rhythm (NSR), and more importantly, Paro. AF from NSR, using short-term single-lead electrocardiogram (ECG) signals. Specifically, we adapted and modified the time-delayed embedding method to minimize incorrect embedding parameter selection and further support to reconstruct proper phase plots of NSR and AF heart dynamics, from MIT-BIH databases. We also examine information-based methods, such as multiscale entropy (MSE) and kurtosis (Kt) for the same purposes. Our results demonstrate that embedding parameter time delay (τ), as well as MSE and Kt values can be successfully used to discriminate between Pers. AF and NSR. Moreover, we demonstrate that τ and Kt can successfully discriminate Paro. AF from NSR. Our results suggest that nonlinear time-delayed embedding method and information-based methods provide robust discriminating features to distinguish both Pers. AF and Paro. AF from NSR, thus offering effective treatment before suffering chaotic Pers. AF.

## 1. Introduction

Atrial fibrillation (AF) is the most common cardiac arrhythmia, and an estimated 2.7–6.1 million people in the United States suffer from AF [1]. AF by itself, is not fatal, but it increases the risk of other fatal cardiac conditions like heart failure and stroke, thus leading to sudden cardiac death [2].

Accurate detection of AF using electrocardiogram (ECG) is challenging. In general, AF is a progressive disease that can be categorized as paroxysmal (Paro. AF), persistent (Pers. AF), and long-lasting AF. Paro. AF is an early stage of the disease with intermittent spontaneously terminated episodes of AF, and Pers. AF and long-lasting AF are characterized by constant presence of AF episodes in the ECG signals. As mentioned in guidelines for the management of AF [3], proper management and treatments should be started no later than the Paro. AF stage. However, this is especially difficult to do because heart rhythms in patients with Paro. AF switches irregularly between normal and abnormal states. Moreover, many previous studies investigating AF detection have not clearly separated between these different types of AF due to the lack of separate opensource Pers. AF and Paro. AF datasets, and only some used their own proprietary datasets separately named Paro. AF and Pers. AF [4]. 

Several studies have attempted to develop improved methods to detect/classify normal sinus rhythm (NSR) and AF (most often Pers. AF.) using ECG signals, based on R-R interval analysis, filtering, spectral analysis, statistical approaches etc. [5,6,7]. Especially, ECG analysis using R-R intervals has been frequently adopted [5,8,9] due to its simplicity and ease of use. However, this analysis does not provide reliable results with respect to Paro. AF discrimination, as it captures regularity of the heart rhythm instead of the entire morphological characteristics in the ECG waveform.

On the other hand, in the past few decades, nonlinear analysis methods have been gaining much attention due to their capability to reveal clinically relevant nonlinear features of the heart’s dynamics, such as fractal dimension [10], correlation dimension [9,11], Lyapunov exponents [8,12], and reconstructed phase space approaches [8,13,14]. As one of the foundations of nonlinear analysis methods, phase space reconstruction using time-delayed embedding [15,16] provides a motivation for nonlinear heart rhythm analysis using a single lead ECG. According to Taken’s theorem [16], the full dynamics of a complex nonlinear system might be reconstructed from a single time series, in the absence of noise. We believe that this method has strong potential to discriminate both Pers. and Paro. AF from NSR by capturing the intrinsic complexity of heart behaviors. Despite these advantages, there is relatively little research directly applying phase space reconstruction using time-delayed embedding method for discriminating between NSR and either types of AF [11,17] and there has been little discussion on discovering inherent properties of nonlinear heart behavior of Pers. AF in short-term signals to improve the discrimination of Paro. AF from NSR.

In addition, several new information-based signal analysis techniques have recently been developed, such as multi-scale entropy (MSE) [7,18,19] and kurtosis (Kt) [20]. These techniques also utilize different cardiac characteristics to uncover the intrinsic complexity of the heart behavior in the normal and abnormal states. These techniques were successfully applied to optical mapping data to identify the pivot points of rotors [20,21,22], and were also used for Pers. AF discrimination from NSR using short time ECG signals [7,23].

**Figure 1:**
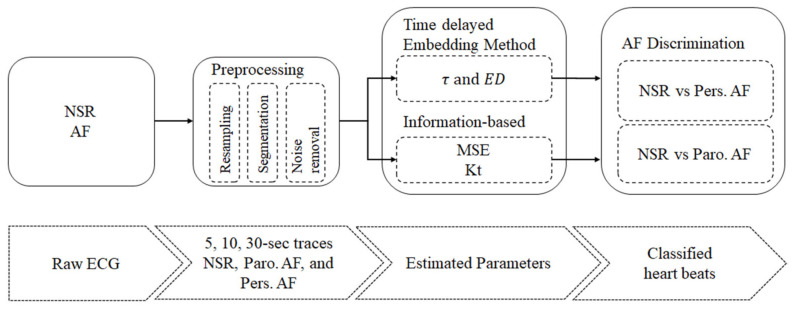
Overview of the process for discriminating AF from normal sinus rhythm (NSR). All NSR and atrial fibrillation (AF) electrocardiogram (ECG) signals first undergo Preprocessing (see Table 1), and the following datasets were created: NSR, paroxysmal AF (Paro. AF), and persistent AF (Pers. AF). Each trace was the cut into 5-, 10-, and 30-second time series, and was analyzed using modified time-delayed embedding method, multiscale entropy (MSE), and kurtosis (Kt) methods to obtain intrinsic characteristics of heart behavior, τ, embedding dimension (ED), MSE, and Kt, specifically. Finally, these parameters were used to differentiate Pers. AF from NSR, and further Paro. AF from NSR.

The aim of this study is to adapt nonlinear time-delayed embedding, MSE, and Kt to discriminate between Pers. AF and NSR, and further Paro. AF and NSR using short-term single-lead ECG signals (see Figure 1). Specifically, we adapt and modify the time-delayed embedding method to reconstruct proper phase plots of NSR and AF heart dynamics. We also examined the robustness of our approaches for AF discrimination using short-term ECG signals of varying lengths: 5, 10, and 30 seconds. 

## 2. Materials and Methods 

### 2.1. Data Description and Preprocessing

ECG traces (2 hours long) of NSR (n=8 patients) and Paro. AF (n=8 patients) were taken from the MIT-BIH NSR and AF databases [24], see Table 1. Note that Paro. AF traces consist of NSR (~82% of NSR) as well as AF episodes that are annotated by clinicians. Following these annotations, we created a Pers. AF database that only consists traces of “true” AF episodes (0% of NSR). All the raw ECG signals were resampled at 128 Hz and filtered via a bandpass of 0.5–40 Hz to remove baseline wander and noise. Each trace was the cut into 5, 10, and 30 seconds long intervals. All analysis in the manuscript is given for 10 seconds traces, and results for 5- and 30-second data are shown in Appendix C. 

**Figure 2:**
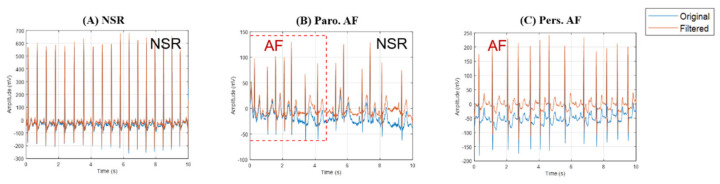
Representative examples of 10-second raw (blue) and filtered (red) traces from single-lead ECG signals: (**A**) NSR, (**B**) Paro. AF, and (**C**) Pers. AF.

Representative examples of 10-second ECG traces are shown in Figure 2: NSR (left), Paro. AF (center), and Pers. AF (right). The original (blue) and filtered signals (red) demonstrate successful removal of the baseline wander in the ECG traces after preprocessing.

### 2.2. Phase Space Reconstruction Using Time-Delayed Embedding Method

Phase space reconstruction was introduced into dynamical system numerically by Packard [25] and formalized by Taken [16]. Taken’s theorem proved that a multi-dimensional state space can be reconstructed from a scalar time series in the absence of noise by calculating time delay (τ) and subsequent embedding dimension (ED). 

Time-delayed embedding approach assumes that the delayed versions of a time series contain information about unobserved state variables. Therefore, lagged versions of the original time series are used to reconstruct a new time series of multiple state vectors. Let us consider a single 10-second single-lead ECG trace (see Section 2.1) as a single time series $\;x\left(t\right)=\left(x_{1},x_{2},\;\ldots ,x_{i},\ldots ,x_{n}\right)$, where n is the number of data points in $x\left(t\right)$. Then a delayed reconstructed state vector is given by $v_{i}=\left(x_{i},x_{i+\tau },x_{i+2\tau },\ldots ,x_{i+\left(d-1\right)\tau }\right)$, where τ is time delay. This vector has intrinsic ED d, which is a minimal required dimension to display the original complex dynamics of the system. The entire reconstructed phase space of the heart dynamics based on a single time series $x\left(t\right)$ can be expressed as the following:

(1) $$V=\;\left[\begin{array}{c} v_{1}\\ v_{2}\\ \vdots \\ v_{i}\\ \vdots \\ v_{n-\left(d-1\right)\tau } \end{array}\right]=\left(\begin{array}{cccc} x_{1} & x_{1+\tau } & \ldots & x_{1+(d-1)\tau }\\ x_{2} & x_{2+\tau } & \ldots & x_{2+(d-1)\tau }\\ \vdots & \vdots & & \vdots \\ x_{i} & x_{i+\tau } & \ldots & x_{i+(d-1)\tau }\\ \vdots & \vdots & & \vdots \\ x_{n-(d-1)\tau } & x_{n-(d-2)\tau } & \ldots & x_{n} \end{array}\right)$$

with $i,\;1\leq i\leq n-\left(d-1\right)\tau$. 

To perform the phase space reconstruction based on time-delayed embedding analysis using a single time series, we execute the following steps: estimate time delay parameter τ, estimate corresponding ED, and finally create reconstruction phase plots. Estimating optimal values for τ and ED are one of the main challenges that we had to address since it entirely depends on the ECG characteristics of NSR, Paro. and Pers. AF.

#### 2.2.1. Estimating τ: Autocorrelation and Mutual Information

The time delay τ can be estimated by measuring the independent τ -separated points in time series. Here, we aim to use two well-known approaches for τ identification to compare their robustness: the computation of autocorrelation (AC) [26] and mutual information (MI) [27]. 

The zero crossing of AC yields the points that minimizes the linear relationship between lagged values of a time series and is calculated by AC function $\Gamma \left(x\left(t\right),\;x\left(t+\tau \right)\right)$:

(2) $$\Gamma \left(x\left(t\right),\;x\left(t+\tau \right)\right)=\frac{\sum_{t=1}^{n-\tau }\left(x_{t}-\bar{x}\right)\left(x_{t+\tau }-\bar{x}\right)}{\sum_{t=1}^{n}{\left(x_{t}-\bar{x}\right)}^{2}},$$

where $\bar{x}$ is the mean of $x\left(t\right)$.

On the other hand, the local minima of MI provide maximum independence including nonlinear correlation, which can be completely blind to certain nonlinear effects in AC with nonlinear dynamics. For this reason, MI function $I\left(x\left(t\right),\;x\left(t+\tau \right)\right)$ has been widely used for selecting τ in nonlinear time series analysis:

(3) $$I\left(x\left(t\right),\;x\left(t+\tau \right)\right)=\underset{i,j}{\sum }p_{i,j}\left(\tau \right)log\frac{p_{i,j}\left(\tau \right)}{p_{i}p_{j}}\;,$$

where $p_{i}$ is the probability that $x\left(t\right)$ is in bin i of the histogram of $x\left(t\right)$, and $p_{i,j}\left(\tau \right)$ is the joint probability that $x\left(t\right)$ is in bin i and $x\left(t+\tau \right)$ is in bin j of the histogram of $x\left(t\right).$

For the effective selection of τ, we identified the RR intervals for each time series $x\left(t\right)$, and calculated AC and MI values up to 1/2 of that period, since further values will be repeated in every beat due to periodicity in heart rhythm (see Appendix A). 

In addition, we improved the traditional way to identify τ, in which only the first zero crossing for AC and the first local minimum for MI are used. Specifically, we identified the first three τ ($\tau_{1},\;\tau_{2}$ and $\tau_{3}$) based on the first three zero crossings for AC and the first three local minima for MI to reduce the risk of improper τ selection. The rationale behind our improvement is the following. In practice, unexpected local minimum dependency values in both AC and MI can be found, resulting from data measurement noise or other small movement of waveforms, and sometimes these values are accidently chosen for the optimal τ. Using this improper τ, even highly correlated coordinates may fail to draw attractors in reconstructed phase plots, misrepresenting the original properties of system dynamics. To avoid this situation, we provide three τ ($\tau_{1},\;\tau_{2}$ and $\tau_{3}$) within a half period of one regular heartbeat and compare their corresponding reconstructed phase plots.

#### 2.2.2. Estimating ED: False Nearest Neighbor Algorithm

After estimating $\tau_{1},\;\tau_{2}$ and $\tau_{3}$, the corresponding embedding EDs were selected using the false nearest neighbor (FNN) algorithm, as described in [28]. This method is based on the principle that the pair of points which are located very near each other at a large enough dimension d will remain close to each other at the dimension d+1. Thus, if the number of neighbors does not change appreciably, then embedding leaves the shape of the attractor unchanged. The neighbors are checked at increasing embedding dimensions until a negligible number of false neighbors are found while moving from dimension d to d+1. In FNN algorithm, the square of the Euclidean distance between d and d+1– dimensional phase space is used to determine the number of neighbors, according to the following equations:

(4) $$Dist_{d}^{2}\left(t,r\right)=\overset{d-1}{\underset{k=0}{\sum }}{\left[x\left(t+k\tau \right)-x^{\left(r\right)}\left(t+k\tau \right)\right]}^{2},$$

(5) $$Dist_{d+1}^{2}\left(t,r\right)=Dist_{d}^{2}\left(t,r\right)+{\left[x\left(t+d\tau \right)-x^{\left(r\right)}\left(t+d\tau \right)\right]}^{2},$$

where r is the number of nearest neighbors. Therefore $ED_{1},ED_{2},$ and $ED_{3}$ were computed using Equations (4) and (5) for the corresponding $\tau_{1},\;\tau_{2}$ and $\tau_{3}$.

If the second term in the right side of Equation (5) is close to zero, then the current dimension d is selected as a minimal required ED to display the original complex dynamics of this system. However, in practical cases, ED is selected when it less than a small threshold (i.e., 1% or 5%), because reaching the zero of FNN is almost impossible or takes too long. The % of FNN value as a function of every increasing d will be used for representing the way to select a minimal required ED:

(6) $$\%ofFNN=\frac{\#ofFNN}{total\;N}\times 100.$$

In this work, $ED_{1},ED_{2},$ and $ED_{3}$ were estimated separately using AC and MI approaches.

#### 2.2.3. Phase Space Reconstruction

Reconstructed phase plots are formed by ED-dimensional coordinates as shifted versions of τ from the single time series $x\left(t\right)$ (as shown in Equation (1)): $\bar{v_{i}}=\left[x_{i}\left(t\right),x_{i}\left(t+\tau \right),\;\ldots ,\;x_{i}\left(t+\left(ED-1\right)\tau \right)\right],\;i=1,\;2,\;\ldots ,\;N-\left(ED-1\right)\tau$. Due to limitations in visualization, we are showing only in 3D plots.

### 2.3. Multiscale Entropy (MSE)

Multiscale entropy measures the unpredictability of a signal by estimating its tendency to repeat itself in long and short sequences, with a greater relative amount of long repeated sequences indicating an orderly and low-entropy signal. However, analysis of short vectors can only investigate self-similarity in small portions of these waveforms. To appreciate large ECG features that may distinguish NSR and AF signals, we implement a moving average filter to ECGs to capture information on a broader time scale and highlight differences in larger-scale dynamics of AF and NSR heart behaviors. 

Prior to analysis, an averaged time series is obtained from the original single time series $x=\left(x_{1},x_{2},\;\ldots ,x_{i},\ldots ,x_{n}\right)$ by applying a moving average filter to x. The size of the moving average is determined by the time scale value $\tau_{MSE}$, creating a time series $z=\left(z_{1},z_{2},\;\ldots ,z_{j},\ldots ,z_{n-\tau_{MSE}}\right)$, such that:

(7) $$z_{j}^{\tau_{MSE}}=\frac{1}{2\tau_{MSE}+1}\overset{2\tau_{MSE}+1}{\underset{i=j}{\sum }}x_{i},$$

where $1\leq j\leq n-\tau_{MSE}$ and i =1, 2, 3, …, n.

The new time series z is then evaluated for self-similarity as was suggested previously [7]. A tolerance range r is determined as one fifth of the standard deviation of the time series, and pairs of vectors within a distance of r of each other are considered a vector match. The relative quantity of long (m+1 length) matches to short (m length) matches is used as a measure of the likelihood of the signal to repeat itself. MSE also employs a delay factor δ, which determines whether compared sequences are constructed from consecutive points or every δ points from the time series z. MSE is therefore evaluated as a function of the time series z:

(8) $$MSE=-ln\;\left(\frac{N_{z}\left(m+1,\delta ,r\right)}{N_{z}\left(m,\delta ,r\right)}\right),$$

where $N\left(\ldots ,\delta ,r\right)$ represents the number of vector matches in z at m and m+1. A proportionally small number of long matches corresponds to a large entropy value. It is expected that AF, having more chaotic dynamics, will have a larger MSE value. In this manuscript, we use $\tau_{MSE}=3$, m=2, δ=1, as was suggested in [7].

### 2.4. Kurtosis

Kurtosis (*Kt*) is a higher-order statistical approach to determine the peakedness of the ECG signal’s amplitude distribution. *Kt* is the fourth moment of statistics, calculated as follows:

(9) $$Kt\;\left(x\right)=E\left\{{\left[\frac{x-E\left(x\right)}{\sigma }\right]}^{4}\right\}=\frac{\mu_{x}^{4}}{\sigma_{x}^{4}}\;,$$

where $E\left(x\right)$ represents the mean value of timeseries x and $\sigma_{x}$ represents the standard deviation. 

### 2.5. Data Analysis

Histograms were constructed by plotting embedding parameters τ, ED, MSE, and Kt values for every 10 second ECG trace from all patients during NSR and Paro. AF (5760 values for each parameter), as well as Pers. AF (5537 values for each parameter). Boxplots were also constructed for each parameter by plotting the average value for each patient, which were calculated as the mean value of approximately 2 hours of ECG traces for NSR, Paro. AF, and Pers. AF. Statistical significance test was performed using one-way ANOVA and the result was judged as significant when the *p*-value ≤0.05 (*).

All the analysis was performed on the single-lead ECG recordings using custom-written MATLAB (MathWorks, Inc., Natick, MA, USA) scripts for all three methods, additionally a CRP MATLAB toolbox [29] is used for the embedding method.

## 3. Results

### 3.1. Optimized Time-Delayed Embedding Method to discriminate Pers. AF from NSR

#### 3.1.1. τ: Pers. AF Discriminator

**Figure 3:**
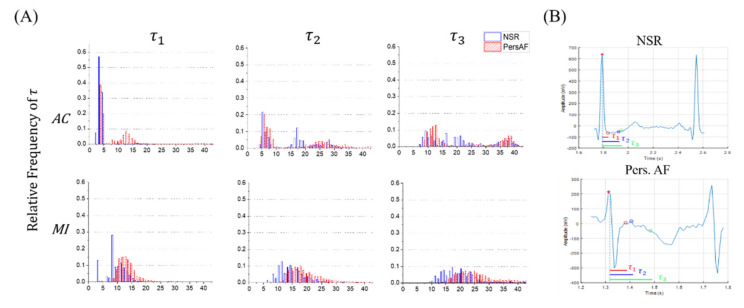
Distribution of the embedding time delay parameter τ between NSR and Pers. AF: (**A**) histograms of $\tau_{1},{\;\tau }_{2},$ and $\tau_{3}$ calculated by autocorrelation (AC) (top) and mutual information (MI) (bottom) and (**B**) representative values of $\tau_{1},{\;\tau }_{2},$ and $\tau_{3}$ in a heartbeat of ECG trace, which are the distances between the two points ($t_{0}$, $\tau_{i=1,\;2,\;3}$). These τ are calculated by MI. From both (**A**) and (**B**), larger τ were observed for Pers. AF.

**Figure A2:**
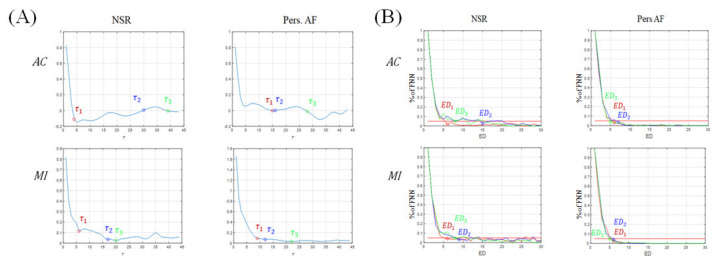
A representative example of embedding parameter estimations. (**A**) Determination of the proper *τ* having the lowest dependency with the original ECG *x*(*t*). Each $\tau_{1},\;\tau_{2}$ and $\tau_{3}$ are calculated by AC and MI methods. (**B**) Determination of the minimal required *ED*. The percentage of FNN (% of FNN) drops convincingly to almost zero (here, 5%) at $ED_{1},\;ED_{2}$ and $ED_{3}$ in the range of 5–15.

To observe the distribution of the embedding delay parameters τ, histograms of $\tau_{1},\;\tau_{2},$ and $\tau_{3}$ (in samples) were obtained for NSR (blue) and Pers. AF (red) for 10-second traces for all subjects (see Figure 3A) both for AC (top panels) and MI (bottom panels) approaches. Figure 3A shows that in general, the mean values of all three τs associated with Pers. AF (red) are higher than those of for NSR, both for MI and AC approaches. Figure 3A also demonstrates that the values of $\tau_{1}$ are in lower ranges, which is due to misidentification of the minimum dependency criteria from noisy signals. In addition, using MI approach leads to smoother and more stable distribution of all three τs for both NSR and Pers. AF, in comparison to AC approach. This suggests that MI approach is less sensitive to nonlinear heart rhythm analysis. Finally, note that the distribution of $\tau_{3}$ (for MI approach) is normally distributed for both NSR and Pers. AF, and is less right-skewed than $\tau_{2}$ distribution. This indicates that $\tau_{3}$ points to the global minimum dependency of a time lag between $\left(x\left(t\right),\;x\left(t+\tau \right)\right)$ in majority of cases (see Appendix A). Therefore, examining all three $\tau_{1},\;\tau_{2},$ and $\tau_{3}$ for MI approach, which are located in the lowest dependency region (see Figure A2), is necessary to investigate all the different intrinsic properties of the heart behavior between Pers. AF from NSR using short-time ECG traces.

Figure 3B compares the representative values of $\tau_{1},\;\tau_{2},$ and $\tau_{3}$ that were obtained for NSR (top) and Pers. AF (bottom). Note that the value of τ was inversely proportional to RR intervals: larger τs were observed for AF (RR≈0.42 s) than for NSR (RR≈0.65 s). Thus, Pers. AF patients have larger τ values. The selected τ also represented different morphological properties between $\left(x\left(t\right),x\left(t+\tau \right)\right)$.

**Figure 4:**
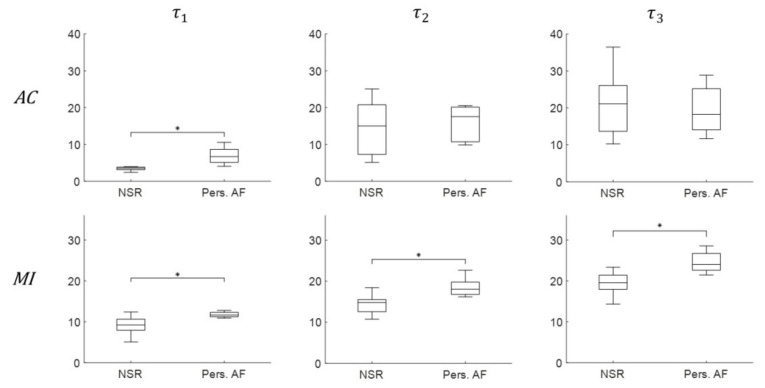
Discrimination boxplots of the embedding parameter $\tau_{1},\;\tau_{2},$ and $\tau_{3}$ using both AC (**top**) and MI (**bottom**) for Pers. AF and NSR. The symbol ‘*’ indicates statistical significance p<0.05 using one-way ANOVA.

To evaluate the ability of τs to discriminate between Pers. AF and NSR presented in Figure 3, the average $\tau_{1},\;\tau_{2},$ and $\tau_{3}$ values of each patient were plotted in Figure 4 both AC (top) and MI (bottom) techniques. Note that MI approach allows for robust discriminations between NSR and Pers. AF for all τ  values (p<0.05), whereas the use of AC approach only leads to $\tau_{1}$ be statistically significant (p<0.05). These findings clearly show that τ calculated using MI approach is a significant and independent discriminator of Pers. AF from NSR. 

#### 3.1.2. ED: Pers. AF Discriminator

**Figure 5:**
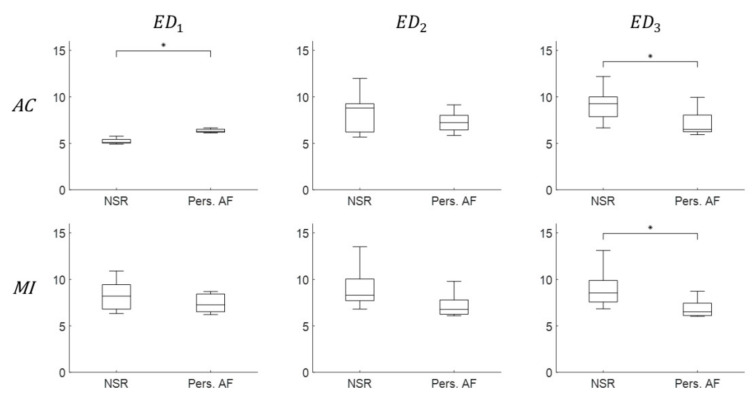
Discrimination boxplots of the embedding parameter $ED_{1},\;ED_{2},$ and $\;ED_{3}$ using both AC (**top**) and MI (**bottom**) for Pers. AF and NSR. ‘*’ indicates statistical significance p<0.05 using one-way ANOVA.

For the investigation of another embedding parameter ED, parameters $ED_{1},\;ED_{2},$ and $\;ED_{3}$ were estimated from corresponding $\tau_{1},\;\tau_{2},$ and $\tau_{3}$ values using FNN algorithm. Figure 5 shows boxplots of the average $ED_{1},\;ED_{2},$ and $\;ED_{3}$ values calculated from NSR and Pers. AF patients for both AC and MI approaches. Note that ED did not offer robust discrimination when compared with τ (see Figure 4). Indeed, only $ED_{1}$ and $ED_{3}$ (calculated using AC approach, p<0.05) and $ED_{3}$ (calculated using MI approaches, p<0.05) were significantly different between Pers. AF from NSR. Note that the variation of ED increases across patients for NSR from $ED_{1}$ to $ED_{3}$, while there are negligible changes for Pers. AF. This arises from the oscillation in FNN calculation as embedding increases (see Figure A2 in Appendix A). Our finding indicates that ED does not robustly discriminate Pers. AF from NSR. 

#### 3.1.3. Phase Space Reconstruction Plots

**Figure 6:**
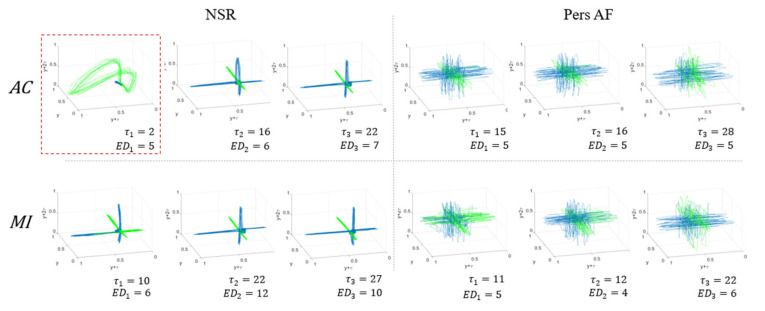
3D visualization of reconstructed phase space plots using the modified time-delayed embedding method. The normalized reconstruction plots with various values of $\tau_{1},{\;\tau }_{2},$ $\tau_{3}$ and corresponding values of $ED_{1},\;ED_{2},$ and $\;ED_{3}$ using both AC (**top**) and MI (**bottom**) approaches are shown to discriminate Pers. AF from NSR. QRS-complexes are represented in green trajectories and the rest of the ECG components are shown in blue.

The full reconstructed phase plots for NSR and Pers. AF ECG traces are shown in Figure 6 for various values of $\tau_{1},\;\tau_{2},$ and $\tau_{3}$ and corresponding values of $ED_{1},\;ED_{2},$ and $\;ED_{3}$. Note that QRS-complexes are represented in green and the rest of the ECG is in blue. Figure 6 demonstrates that the reconstructed trajectories of heart behaviors were visually distinguishable between NSR and Pers. AF patients. The reconstructed plots of NSR show trajectories of regular heartbeats in three orthogonal directions. The green closed trajectory reflects QRS complexes and the P-waves in ECG traces are in near the origin. The trajectories for Pers. AF also consist of similar orthogonal directions, but their trajectories have larger variance, due to the properties of Pers. AF, such as absence or distortion of P-wave, faster RR interval, prolonged QRS complex, noisy T-wave, etc. 

The impact of misidentified $\tau_{1}$ is presented in Figure 3A. This is shown as an example of reconstructed plots of NSR with an inadequate choice of $\tau_{1}$ (red box) and better choices of $\tau_{2}$ and $\tau_{3}$ (NSR from AC). This small $\tau_{1}$ (τ=2) reproduces a different shape of attractor compared to that of $\tau_{2}$ and $\tau_{3}$. The trajectories constructed by too small $\tau_{1}$ do not preserve the original heart dynamics because of misidentified minimum dependencies, and they eventually result in reconstruction phase plots with no consistency. Thus, it is further evidence that our strategy of estimating all $\tau_{1},{\;\tau }_{2},$ and $\tau_{3}$, helps to obtain proper and consistent trajectories of heart behaviors to discriminate between NSR and Pers. AF. 

Figure 6 also shows that the reconstructed phase plots between NSR and Pers. AF have distinguishable trajectories for similar ED values. Note that reconstructed phase plots visually discriminate Pers. AF from NSR, which appropriate embedding parameters τ and ED, despite ED not being a robust independent discriminator. Here, we are displaying only in 3D; however, these differences will be more prominent in the ED-dimensional (higher dimensional) representation, by revealing the intrinsic NSR and AF properties.

### 3.2. Information-Based Methods to Discriminate Pers. AF from NSR

As before in the modified embedding method, we investigated and confirmed the ability of two information-based methods MSE and Kt, for discriminating AF’s intrinsic properties from NSR. 

#### 3.2.1. MSE

**Figure 7:**
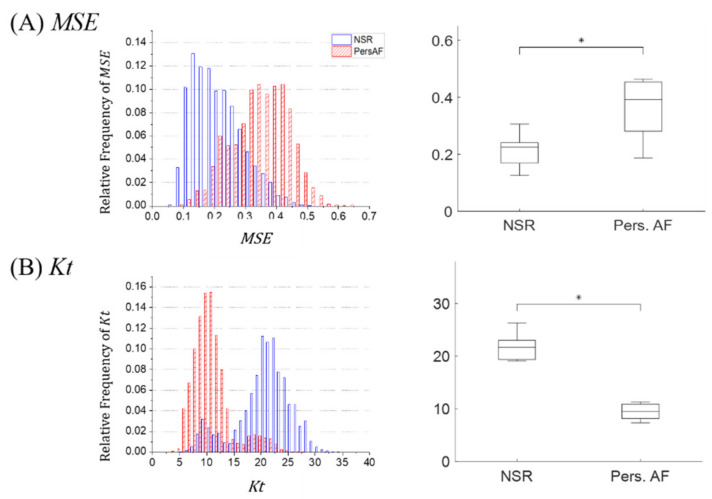
Information-based method analysis: Histograms and boxplots between NSR (blue) and Pers. AF (red) (**A**) MSE (**B**) Kt. ‘*’ indicates statistical significance p<0.05 using one-way ANOVA.

We employ MSE approach to estimate the unpredictability of signals in NSR and Pers. AF ECG traces. The comparisons of a histogram and a boxplot between NSR (blue) and Pers. AF (red) are shown in Figure 7A respectively. As shown in these distributions, significantly higher values of MSE were observed for Pers. AF compared to those of NSR. Our results indicate that AF ECG traces include less matched templates at successive lengths, due to the presence of uncoordinated electrical activity in the atria. MSE approach successfully discriminates between Pers. AF and NSR by utilizing the unpredictability of these ECG signals.

#### 3.2.2. Kt

To perform discrimination between NSR and Pers. AF, we additionally apply Kt as a higher-order statistical approach to emphasize the irregular properties of AF signals. As shown in a histogram and a boxplot for Pers. AF (red) and NSR (blue) in Figure 7B respectively, Kt values for NSR were significantly different compared to those of Pers. AF, with higher variations for NSR. The lower Kt value of AF was due to the wide range of amplitudes found in Pers. AF traces, which flattens the amplitude distribution and reduces the measured peakedness. 

### 3.3. Paro. AF discrimination

**Figure 8:**
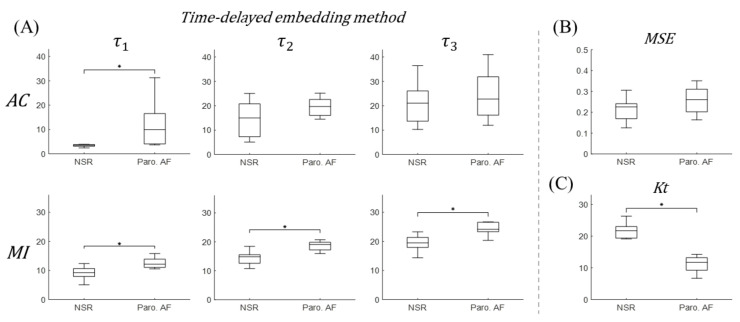
Discrimination boxplots of embedding parameter τ ($\tau_{1},{\;\tau }_{2},$ and $\tau_{3}$) (**A**), MSE (**B**), and Kt (**C**) values between Paro. AF and NSR. NSR and Paro. AF are significantly different in all τ calculated using MI approach and Kt values. ‘*’ indicates statistical significance p<0.05 using one-way ANOVA.

Finally, Figure 8 shows the feasibility of our discriminating embedding parameters τ, and MSE, Kt values in information-based approaches to discriminate Paro. AF from NSR using short time single-lead ECG traces. The boxplots of embedding parameters $\tau_{1},{\;\tau }_{2},$ and $\tau_{3}$ calculated using AC and MI approaches, as well as MSE and Kt values are shown in Figure 8. This clearly demonstrates that the average of all $\tau_{1},{\;\tau }_{2},$ and $\tau_{3}$ values of each patient calculated from MI (bottom in (A)), were robust discriminators between NSR and Paro. AF with statistical significance p<0.05. In addition, the distinguishably lower average Kt values were observed for Paro. AF compared to that for NSR with p<0.05. Therefore, time delay embedding parameter τ using MI approach and Kt values are significant and independent discriminators of Paro. AF from NSR. 

## 4. Discussion

In this study, we first evaluated our recently developed methods, time-delayed embedding method, MSE, and Kt, to discriminate Pers. AF from NSR using short time single-lead ECG signals. Next, the feasibility of discriminating Paro. AF from NSR was assessed. The main results are summarized as follows: (1) The embedding time delay parameter $\tau_{1},{\;\tau }_{2},$ $\tau_{3}$ using MI approach, MSE, and Kt values were able to discriminate Pers. AF from NSR. (2) The embedding parameter $\tau_{1},{\;\tau }_{2},$ $\tau_{3}$ using MI approach, and Kt values were successfully able to discriminate Paro. AF from NSR. (3) The procedure for discrimination between NSR and AF is based on time-delayed embedding method using MI approach. (4) The conventional procedure to select time delay parameter (choosing $\tau_{1}$) may return improper reconstructed plots. (5) Higher τ, MSE, and lower Kt in Pers. AF indicate its chaotic nature compared to NSR. The critical value of our study is that nonlinear time-delayed embedding method and information-based methods provide robust discriminating features to distinguish Paro. AF from NSR. 

In many previous studies of AF detection, Paro. AF and Pers. AF, which have different critical information, were not considered separately [8,9]. Identification of AF episodes is of primary focus rather than detection of the intrinsic conditions of heart dynamics in the presence of NSR and mixed AF in Paro. AF patients. However, our nonlinear analysis methods have great capability to discriminate Paro. AF from NSR even in the presence of approximately 82% NSR. By capturing significantly differentiable features of embedding parameter τ and Kt between AF and NSR, clear identification of Paro. AF from NSR is feasible. This makes our methods contribute a step towards the development of more robust Paro. AF detection approaches and treatment of AF before suffering chaotic Pers. AF. 

### 4.1. Benefits of Time-Delayed Embedding Method

Due to its ability to rebuild the full dynamics of a complex nonlinear system from a single observed time series for an infinitely long noise-free observation in theory, phase space reconstruction using time-delayed embedding also has strong potential for nonlinear system analysis in practical cases. In time-delayed embedding method, estimating optimal values for the parameters τ and ED is one of the main challenges. Although Taken’s theorem suggests that any τ value can be chosen (excluding periodicities) and makes equivalent state space of an observed system in the absence of noise [30], in practice, the choice of τ affects the ability to reconstruct equivalent phase plot, which should preserve geometrical invariants of the original system. Thus, while there has been some theoretical discussion of what constitutes an optimal τ and optimal ED [31], there are no universal strategies so far for implementation of time-delayed embedding in practical applications, such as AF discrimination. 

To address this, our time-delayed embedding strategy suggests one possible optimal process that can be applied to AF discrimination problem, by selecting three embedding parameters and plotting the distinguishable reconstructed phase plots between NSR and Pers. AF. Particularly, we provide three τ ($\tau_{1},{\;\tau }_{2}$ and $\tau_{3}$) within a half period of one regular heartbeat and compare their corresponding reconstructed phase plots. This minimizes the selection of incorrect embedding parameters and guarantees the ability to reconstruct phase portraits of the original heart dynamics to discriminate AF from NSR. In Appendix A, the cyclic behavior of heartbeats and an improper case of too small of τs in MI dependency measurements for estimating τ parameters are addressed. It was the motivation of our strategy and the evidence of the need for multiple τs and of their searching range for application in AF discrimination.

The embedding parameter τ calculated using MI approach was an independent discriminator for both Pers. AF and Paro. AF. It has strong possibility in the correlation between τ and QRS-complex duration. In Figure 3B NSR case, the length of $\tau_{1}$ is approximately represented from the R-peak ($t_{0}$) to the end of the QRS-complex, which is the minimal duration where its morphological characteristics can be changed in a single beat of ECG waveform. This duration of $\tau_{1}$ for Pers. AF has been prolonged compared to $\tau_{1}$ in NSR, and this is in consistent with the slightly prolong duration of QRS-complex for abnormal rhythms in general (Normal: 80–100 ms, intermediate: 100–120 ms, abnormal: >120 ms). Thus, this embedding parameter τ may reflect the RS duration (the half QRS-complex duration). As shown in our results, τ larger than 5 (in samples, 40–50 ms in times) for NSR and higher value of AF were observed. 

### 4.2. Benefits of Information-Based Methods

MSE and Kt values themselves were significant discriminating factors, discriminating Pers. AF and NSR through analysis of self-similarity and peakedness of ECG trace amplitude respectively. 

The MSE method yield a more meaningful approach than conventional entropy measurements like sample entropy for short-term physiological signals. MSE was applied on the short ECG traces and clearly distinguishes between NSR and Pers. AF. In Appendix B, the value of entropy for the coarse-grained time series is plotted as a function of time scales. The MSE calculation yields consistent findings that the entropy measure from Pers. AF has significantly higher value which indicates more unpredictable time series. We also find clear reverse patterns from the increasing entropy measure of NSR and the decreasing measure of Paro. AF, which were not significantly different with a single time scale factor in Figure 7A. In this respect, analyzing MSE for a range of time scales is valuable for evaluating the irregularity of ECG signals to discriminate AF from NSR.

Kt highlights the differences in the signal amplitude distributions. The regularity of NSR produces narrower and taller amplitude distributions (e.g., R-, T- and P-peaks), resulting in a significantly larger Kt value compared to the wider and flatly distributed signal amplitudes of AF patients. This reduced peaknedness in the amplitude distribution of AF ECG traces helps distinguish both Pers. AF and Paro. AF from NSR and makes Kt an independent discriminator.

Decreased Kt values in Pers. AF may be partially explained by the absence of P-waves. As periodic P-wave are replaced by numerous, lower amplitude aperiodic waves in each cycle in the ECG, the amplitude distribution of the analyzed signal will change considerably. The periodic/regular P-waves in ECG contributes to narrow and tall amplitude distributions in NSR, as the amplitude falls in a smaller interval. But in the case of the AF traces the absence of regular P-waves changes the range of amplitude distributions to be more random. This in turn causes the distribution to be flatter and wider, correspondingly reducing the Kt values.

The decreased Kt values for Paro. AF may be related to a number of electrophysiological changes involved with the incidence and perpetuation of AF. In response to fast atrial rhythms in AF episodes, atria can undergo a number of changes such as lowered conduction, due to calcium ($Ca^{2+}$) release [32]. ECG readings from this slow-conducting atria display a prolonged P-wave, which is a biomarker to predict AF onset [33]. This prolonged P-wave has a different amplitude morphology and replaces part of the PR segment. This can reduce the peakedness of the amplitude distribution and the corresponding Kt value. These changes may manifest in ECG recordings even outside of AF episodes, resulting in significant changes to Paro. AF Kt values and not only Pers. AF.

Based on these clear differences in MSE and Kt values, our information-based methods have strong potential as AF discriminator that utilize unpredictability and amplitude distribution, respectively. 

### 4.3. Validation of Three Nonlinear Methods with Short Length of ECG

To evaluate the robustness of our three methods with respect to length of short-term ECG signals to discriminate Pers. AF, Paro. AF, and NSR, we examined their performance for different lengths of ECG traces from short (5 s) to long (30 s), and representative valid results from 5-, 10-, and 30-second traces were presented. Similar to results in 10-second ECG traces, τ, MSE, and Kt values were able to discriminate between NSR and Pers. AF, and τ and Kt performed well for discriminating Paro. AF from NSR for all the tested time lengths. More detailed information on methods and results are presented in Appendix C.

### 4.4. Influence of Noise Removal 

It is essential to remove noise before the implementation of the three nonlinear approaches on the ECG traces. Noise removal can be performed using various methods such as adaptive least mean squares (LMS) filtering, Kalman filtering, wavelet decomposition, bandpass filtering, etc. [34,35]. The complexity of the noise removal techniques increases depending on the purpose of the removal level of noise present. In our work, a bandpass filter that restricts the signals within the physiological frequency range of 0.5 Hz to 40 Hz for AF identification is used as the minimal level of noise removal.

### 4.5. Limitations

One of the main limitations of this work is the limited data set used. In the future, larger data sets can be integrated to show and analyze both Paro. AF and Pers. AF separately. In order to confirm the potential use of our approaches, validation in datasets from more subjects as well as different datasets is necessary to reduce bias and uncertainty in both Pers. and Paro. AF discrimination. Another limitation is that the reconstructed phase plot cannot visualize the full attractor of the original system dynamics, especially where the state space is high dimensional (or even infinite) or where the dynamics have spatio-temporal properties. More quantitative measures using the reconstructed phase space must be explored to automate the discrimination. Despite all these limitations, our work highlights the potential utility in identifying which single technique or combination of techniques is necessary to accurately discriminate AF from NSR.

## 5. Conclusions

We have presented three nonlinear approaches to discriminate between patients with AF and NSR using a short time single-lead ECG. The proposed strategy using time-delayed embedding method and Kt provided highly robust and reliable discriminating features to distinguish Paro. AF from NSR. These results motivate improved methods using single or combining our techniques to more accurately characterize Paro. AF and further other arrhythmias, and they offer a potential clinical utility in effective treatment before the onset of severe Pers. AF or long-lasting AF as well as diagnosis and prognosis of life-threatening heart disease for real-time ECG monitoring. 

## Figures and Tables

**Table 1:** Data Acquisition Information and Preprocessing Procedure

		NSR	Paro. AF	Pers. AF
Database		MIT-BIH NSR	MIT-BIH AF	MIT-BIH AF
No. of Patients		8 (100% of NSR)	8 (~82% of NSR)	8 (0% of NSR)
Sampling Rate, Hz		128	250 → 128	250 → 128
No. of 10-sec segments	total	5760	5760	5537
	per patient	720	720	20, 33, 134, 1953, 304, 1733, 584, 776
Filtering		Baseline wander removal by bandpass filter [0.5, 40] Hz		

**Figure A1:**
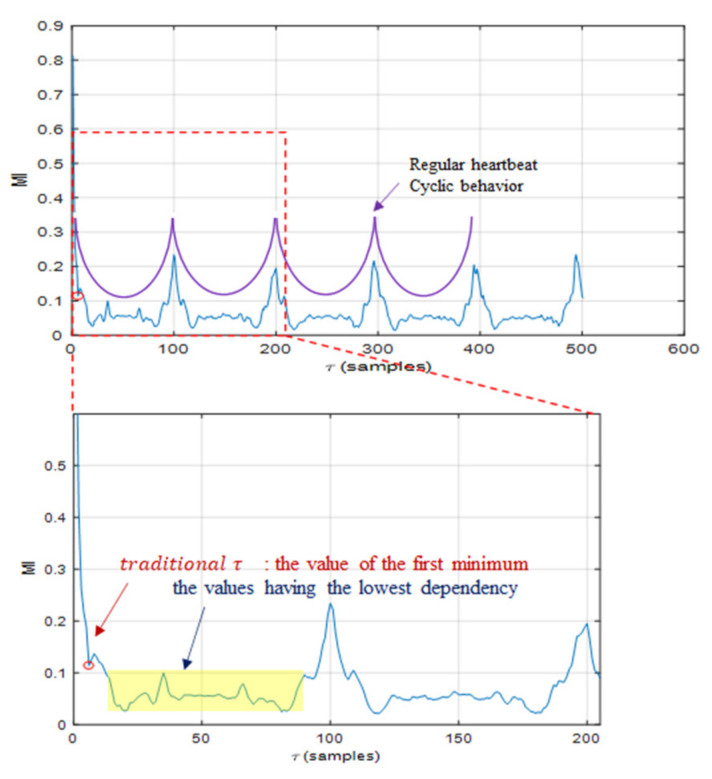
Motivation to select multiple τ. The cyclic behavior of heartbeats and a misidentified τ in MI dependency measurements for estimating τ parameters are addressed. Our strategy provides three τ ($\tau_{1},{\;\tau }_{2}$ and $\tau_{3}$) with in a half period of one regular heartbeat and compares their corresponding reconstructed phase plots, in order to reduce the risk of misidentified τ.

**Figure A3:**
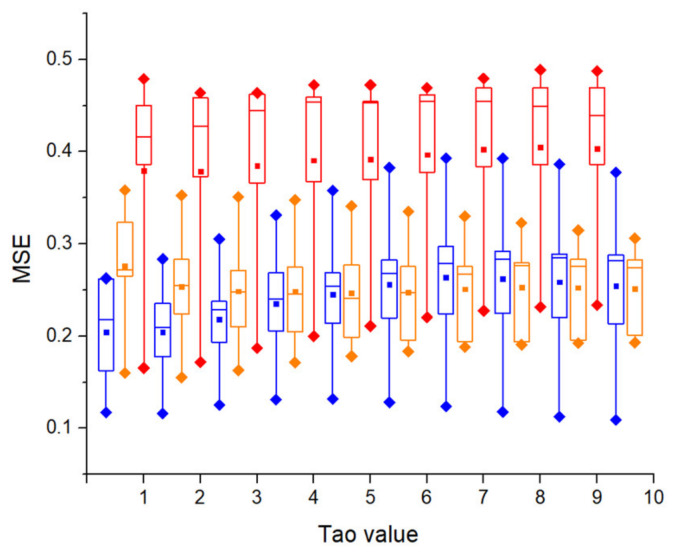
MSE analysis derived from NSR (blue), Paro. AF (orange), and Pers. AF (red). Values are given as average values of each patients (n = 5).

**Figure A4:**
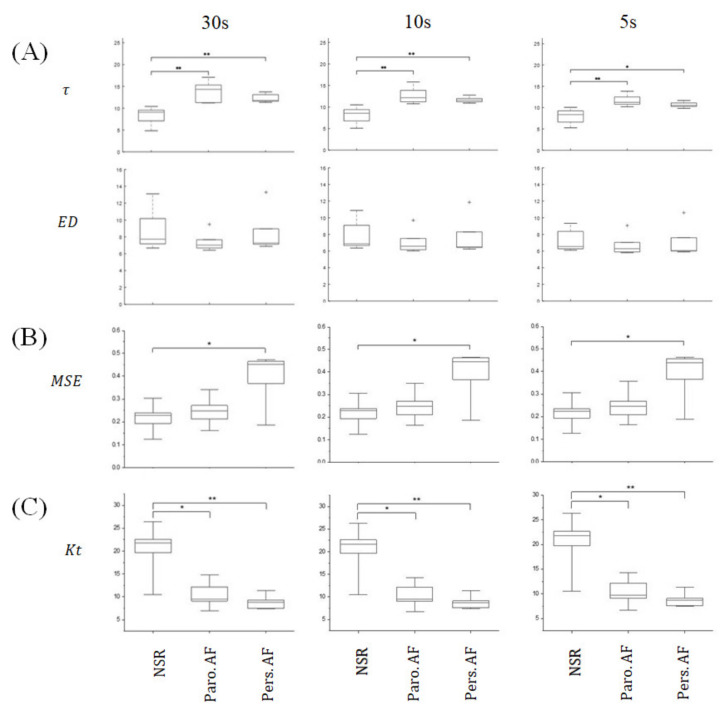
The robustness of AF discriminators. The similarities between nonlinear methods with different length of ECG traces (30 s, 10 s, and 5 s) are shown. Estimating (**A**) $\tau_{1}$ and their corresponding $ED_{1}$ from the modified embedding method, (**B**) MSE, and (**C**) Kt for all groups (NSR, Paro. AF, and Pers. AF). All the results with different lengths of signals are consistent.

## Appendix A. Motivation to Select Multiple Embedding Time Delay *τ*

We improved the traditional way to identify τ, in which only the first zero crossing for AC and the first local minimum for MI are used to select the minimum dependency between original and shifted time series. In practice, the signals analyzed are not ideal and have minimal noise and disturbances, that results in misidentification of local minimum dependency values in both AC and MI, thus resulting in improper τ. Using this improper τ, even highly correlated coordinates may fail to draw attractors in reconstructed phase plots, misrepresenting the original properties of system dynamics.

The cyclic behavior of heartbeats and a misidentified τ in MI dependency measurements for estimating τ parameters are addressed in Figure A1. This dependency graph has peaks almost every 100 samples (~0.78 s) which are exactly matched with single regular heartbeat of NSR patients. If we select τ traditionally as a value of the first local minimum, sometimes it provides a distorted and disformed phase plot, which could not preserve the intrinsic properties of heart behavior (see in Figure 5 the first graph, $\tau_{1}$ calculated by AC). However, there are more stable lowest dependency regions (yellow highlighted) in every regular heartbeat. Thus, selection of multiple local minima for MI within the lowest dependency region and comparing all their reconstructed plots will reduce error in the reconstructed phase plot, providing a possible optimal solution.

Specifically, we identified the first three τ ($\tau_{1},\;\tau_{2}$ and $\tau_{3}$) based on the first 3 zero crossings for AC and the first three local minima for MI to reduce the risk of improper τ selection. For the effective selection of τ, we identified the period RR for each time series $x\left(t\right)$, and calculated AC and MI values up to 1/2 of that period, since further values will be repeated in every beat due to periodicity in heart rhythm (see Figure A1).

Figure A2 shows a representative example of time-delayed embedding parameter estimations to discriminate Pers. AF from NSR. The visualization of estimating $\tau_{1},\;\tau_{2}$ and $\tau_{3}$ is in Figure A2A and estimating $ED_{1},\;ED_{2}$ and $ED_{3}$ is in Figure A2B.

First, as shown in Figure A2A, the measurement of dependency between the original 10-second ECG $x\left(t\right)$ and the shifted segment $x\left(t+\tau \right)$ are calculated from AC (top) and MI (bottom) methods respectively. Estimated $\tau_{1},\;\tau_{2}$ and $\tau_{3}$ are marked on each graph, which are the first three zero crossings in AC and the first three local minima in MI. These values provide the possible optimal time delay τ, which has the lowest dependency between ($x\left(t\right),\left(t+\tau \right)$), producing the minimum time lag to rebuild the reconstructed phase plots.

As presented in Figure A1, misidentified $\tau_{1}$ can be found in MI calculation for NSR. Here, $\tau_{1}$ is absolutely in the local minima, however, the dependency measure still has been decreasing, thus more stable minimum can be found near $\tau_{2}$ and $\tau_{3}$. Relatively more cases of misclassified $\tau_{1}$ were found for NSR than Pers. AF cases.

Then, to estimate the minimal required ED, the percentage of FNN (% of FNN) values are calculated as a function of embedding dimension (ED). In Figure A2B, minimally required $ED_{1},\;ED_{2}$ and $ED_{3}$ were selected, which are the points that FNN drops convincingly close to zero (<5%). Due to the fluctuations of % of FNN values for NSR, ED’s cutting point cannot be zero. Note that much higher oscillations in % of FNN for NSR can be found, leading the increasing variations in ED across patients for NSR in Figure 4 boxplots. On the other hand, surprisingly all ED for Pers. AF satisfied within the ED range of 5–8, further converged to zero at around 10. For that reason, the complexities of NSR and Pers. AF cannot be directly compared, and ED is not preferable to use for AF discrimination from NSR independently.

## Appendix B. MSE

In physiological data, many variations of MSE [7,18,19] has been developed to measure the complexity of time series for a range of scales. In this study, we examined the value of MSE in 10-second ECG traces for analysis of discriminating Paro. AF from NSR. In Figure A3, the value of entropy (Sample Entropy) for the coarse-grained time series is plotted in function of the time scale. Pers. AF (red), Paro. AF (orange), and NSR (blue).

In Figure A3, we observed three different types of behaviors: (1) For all time scales, a higher value of entropy was observed for ECG traces from Pers. AF, containing more complex structures than NSR traces. (2) The entropy measure for NSR increases on small time scales and then stabilizes to a constant value. This is due to the smoothing effect of the dynamic moving average filter that reduces the abrupt morphological changes of the signal. As a result, fewer matches are detected in the signal, which is inherently regular. (3) The entropy measure for Paro. AF decreases on small time scales and then stabilizes to a constant value with decreasing variances. Of note, for scale one, Paro. AF has a higher entropy than NSR, but for scales > 4, NSR and Paro. AF MSE values become indistinguishable. These reductions in MSE at higher time scales for Paro. AF indicate that their signals have similar regularity when smoothed by a moving average filter.

Our results seem to suggest that Paro. AF electrical activity is inherently less regular than NSR, even outside of arrhythmic episodes. This may be a result of heterogenous conductance and $I_{C_{a}}$ slowing that occurs in response to fast atrial pacing [32]. Uneven modifications to electrical activity could result in changes that are detected at lower time scales but removed at higher scales.

The MSE method yields consistent findings that the entropy measure from Pers. AF is significantly higher value, indicating more unpredictable time series. We also found clear reverse patterns from the increasing entropy measure of NSR and the decreasing measure of Paro. AF, which were not distinguishable in a single time scale factor ($\tau_{MSE}=3$) in Figure 8B. In this respect, the MSE method appears to yield a more meaningful approach than conventional entropy measurements.

## Appendix C. Validation of Three Nonlinear Methods with Short Length of ECG

To evaluate the robustness of our three methods with respect to length of short-term ECG signals to discriminate Pers. AF, Paro. AF, and NSR, we examined their performance for different lengths of ECG traces from short (5 s) to long (30 s). The same process as described previously in the method section, was followed for each short-term signal. The representative discriminating results from 30, 10, and 5 seconds are presented in the form of boxplots in Figure A4. Properties are similar between all tested durations for the embedding parameter, τ, MSE, and Kt values as observed in previous results. Similarly, τ, MSE, and Kt values were able to discriminate between NSR and Pers. AF, and τ and Kt performed well for discriminating Paro. AF from NSR for all the tested time lengths.

## References

[1] Atrial Fibrillation: Facts about AFib. https://www.cdc.gov/heartdisease/atrial_fibrillation.htm.

[2] Biosense Webster EMEA (2018). The Burden of Atrial Fibrillation: Understanding the Impact of the New Millennium Epidemic across Europe.

[3] Camm A.J., Kirchhof P., Lip G.Y., Schotten U., Savelieva I., Ernst S., Van Gelde I.C., Al-Attar N. (2010). Guidelines for the management of atrial fibrillation: The Task Force for the Management of Atrial Fibrillation of the European Society of Cardiology (ESC). Eur. Heart J..

[4] Park J., Lee C., Leshem E., Blau I., Kim S., Lee J.M., Hwang J.-A., Choi B., Lee M.-H., Hwang H.J. (2019). Early differentiation of long-standing persistent atrial fibrillation using the characteristics of fibrillatory waves in surface ECG multi-leads. Sci. Rep..

[5] Tateno K., Glass L. (2001). Automatic detection of atrial fibrillation using the coefficient of variation and density histograms of RR and ΔRR intervals. Med. Biol. Eng. Comput..

[6] Arunachalam S.P., Annoni E.M., Kapa S., Mulpuru S.K., Friedman P.A., Tolkacheva E.G. (2017). Multiscale frequency technique robustly discriminates normal sinus rhythm and atrial fibrillation on a single lead electrocardiogram C3. 54th Annual Rocky Mountain Bioengineering Symposium, RMBS 2017 and 54th International Biomedical Sciences Instrumentation.

[7] Arunachalam S.P., Kapa S., Mulpuru S.K., Friedman P.A., Tolkacheva E.G. (2018). Improved Multiscale Entropy Technique with Nearest-Neighbor Moving-Average Kernel for Nonlinear and Nonstationary Short-Time Biomedical Signal Analysis. J. Healthc. Eng..

[8] Perc M. (2005). Nonlinear time series analysis of the human electrocardiogram. Eur. J. Phys..

[9] Nayak S.K., Bit A., Dey A., Mohapatra B., Pal K. (2018). A review on the nonlinear dynamical system analysis of electrocardiogram signal. J. Healthc. Eng..

[10] Kiani K., Maghsoudi F. (2019). Classification of 7 Arrhythmias from ECG Using Fractal Dimensions. J. Bioinforma. Syst. Biol..

[11] Mehta C., Miller M. (2007). Chaos Analysis for EKG Time Series Data.

[12] San-Um W., Ketthong P. The quantitative analysis of nonlinear behaviors of arrhythmia through Lyapunov Exponents. Proceedings of the 7th 2014 Biomedical Engineering International Conference.

[13] Al-Fahoum A.S., Qasaimeh A.M. (2013). A practical reconstructed phase space approach for ECG arrhythmias classification. J. Med. Eng. Technol..

[14] Wallot S., Mønster D. (2018). Calculation of Average Mutual Information (AMI) and false-nearest neighbors (FNN) for the estimation of embedding parameters of multidimensional time series in matlab. Front. Psychol..

[15] Whitney H., Eells J., Toledo D. (1992). Collected Papers of Hassler Whitney.

[16] Takens F., Rand D., Young L.-S. (1981). Detecting strange attractors in turbulence BT—Dynamical Systems and Turbulence, Warwick 1980.

[17] Hundewale N., Arabia S. (2012). The application of methods of nonlinear dynamics for ECG in Normal Sinus Rhythm. Int. J. Comput. Sci. Issues.

[18] Costa M., Goldberger A.L., Peng C.-K. (2002). Multiscale entropy analysis of complex physiologic time series. Phys. Rev. Lett..

[19] Wu S.-D., Wu C.-W., Lee K.-Y., Lin S.-G. (2013). Modified multiscale entropy for short-term time series analysis. Phys. A Stat. Mech. Appl..

[20] Arunachalam S.P., Annoni E.M., Mulpuru S.K., Paul A., Tolkacheva E.G. Kurtosis as a Statistical Approach to Identify the Pivot Point of the Rotor Kurtosis as a Statistical Approach to Identify the Pivot Point of the Rotor. Proceedings of the 2016 38th Annual International Conference of the IEEE Engineering in Medicine and Biology Society.

[21] Annoni E., Friedman P.A., Annoni E.M., Arunachalam S.P., Kapa S., Mulpuru S.K., Paul A. (2017). Novel Quantitative Analytical Approaches for Rotor Identification and Associated Implications for Mapping Novel Quantitative Analytical Approaches for Rotor Identification and Associated Implications for Mapping. IEEE Trans. Biomed. Eng..

[22] Arunachalam S.P., Annoni E.M., Mulpuru S.K., Friedman P.A., Tolkacheva E.G. (2016). Novel multiscale frequency approach to identify the pivot point of the rotor. J. Med. Device..

[23] Poigai Arunachalam S., Annoni E.M., Kapa S., Mulpuru S.K., Friedman P.A., Tolkacheva E.G. Robust Discrimination of Normal Sinus Rhythm and Atrial Fibrillation on ECG Using a Multiscale Frequency Technique. Proceedings of the 2017 Design of Medical Devices Conference DMD 2017.

[24] Goldberger A.L., Amaral L.A.N., Glass L., Hausdorff J.M., Ivanov P.C.H., Mark R.G., Mietus J.E., Moody G.B., Peng C.-K. (2003). PhysioBank, PhysioToolkit, and PhysioNet: Components of a New Research Resource for Complex Physiologic Signals. Circulation.

[25] Packard N.H., Crutchfield J.P., Farmer J.D., Shaw R.S. (1980). Geometry from a time series. Phys. Rev. Lett..

[26] Kantz H., Schrieber T. (2003). Nonlinear Time Series Analysis.

[27] Fraser A.M., Swinney H.L. (1986). Independent coordinates for strange attractors from mutual information. Phys. Rev. A.

[28] Kennel M.B., Brown R., Abarbanel H.D.I. (1992). Determining embedding dimension for phase-space reconstruction using a geometrical construction. Phys. Rev. A.

[29] Marwan N., Thiel M., Nowaczyk N.R. (2002). Cross recurrence plot based synchronization of time series. arXiv.

[30] Chelidze D. Delay Coordinate Embedding. https://personal.egr.uri.edu/chelidz/documents/mce567_Chapter_7.pdf.

[31] Casdagli M., Eubank S., Farmer J.D., Gibson J. (1991). State space reconstruction in the presence of noise. Phys. D Nonlinear Phenom..

[32] Shiroshita-Takeshita A., Brundel B.J.J.M., Nattel S. (2005). Atrial fibrillation: Basic mechanisms, remodeling and triggers. J. Interv. Card. Electrophysiol..

[33] Caldwell J., Koppikar S., Barake W., Redfearn D., Michael K., Simpson C., Hopman W., Baranchuk A. (2014). Prolonged P-wave duration is associated with atrial fibrillation recurrence after successful pulmonary vein isolation for paroxysmal atrial fibrillation. J. Interv. Card. Electrophysiol..

[34] Lin H.-Y., Liang S.-Y., Ho Y.-L., Lin Y.-H., Ma H.-P. (2014). Discrete-wavelet-transform-based noise removal and feature extraction for ECG signals. Irbm.

[35] Haritha C., Ganesan M., Sumesh E.P. A survey on modern trends in ECG noise removal techniques. Proceedings of the 2016 International Conference on Circuit, Power and Computing Technologies (ICCPCT).

